# Investigation of a spontaneous mutant reveals novel features of iron uptake in *Shewanella oneidensis*

**DOI:** 10.1038/s41598-017-11987-3

**Published:** 2017-09-18

**Authors:** Ziyang Dong, Shupan Guo, Huihui Fu, Haichun Gao

**Affiliations:** Institute of Microbiology and College of Life Sciences, Zhejiang University, Hangzhou, Zhejiang 310058 China

## Abstract

*Shewanella oneidensis* is among the first and the best studied bacteria capable of respiring minerals as terminal electron acceptors (EAs), including a variety of iron ores. This respiration process relies on a large number of *c*-type cytochromes, which *per se* are iron-containing proteins. Thus, iron plays an essential and special role in iron respiration of *S. oneidensis*, prompting extensive investigations into iron physiology. Despite this, we still know surprisingly little about the components and characteristics of iron transport in this bacterium. Here, we report that TonB-dependent receptor PutA (SO_3033) is specific to the siderophore-mediated iron uptake. Although homologs of PutA are abundant, none of them can function as a replacement. In the absence of PutA, *S. oneidensis* suffers from an iron shortage, which leads to a severe defect in production of cytochrome *c*. However, proteins requiring other types of cytochromes, such as *b* and *d*, do not appear to be significantly impacted. Intriguingly, lactate, but not other carbon sources that are routinely used to support growth, is able to promote iron uptake when PutA is missing. We further show that the lactate-mediated iron import is independent of lactate permeases. Overall, our results suggest that in *S. oneidensis* the siderophore-dependent pathway plays a key role in iron uptake when iron is limited, but many alternative routes exist.

## Introduction

Iron (Fe), one of the most abundant elements in Earth’s crust, is cycled primarily between the reduced ferrous Fe(II) and the oxidized ferric Fe(III) forms by virtually all living organisms^1^. By mediating redox reactions, iron participates in an array of biological processes as an extremely versatile prosthetic component for proteins^2^. Despite its abundance, iron acquisition remains a challenge to life in general, because in aerobic environments iron exists in the extremely insoluble ferric form. To overcome this, bacteria evolve delicate systems enabling iron, in various forms, to be scavenged from the surroundings. Among them, siderophore-dependent iron acquisition is particularly important. Siderophores are high-affinity chelators of Fe(III); when secreted into the environment they interact with iron to form a ferri-siderophore complex^3^. In Gram-negative bacteria, subsequent uptake of the complexes mainly relies on TonB-dependent receptors in the outer membrane (OM) and the energy-transducing TonB-ExbB-ExbD system spanning the inner membrane (IM) and the periplasm^4^. An ABC transporter is then responsible for Fe(III)-siderophore translocation across IM into the cytoplasm, where Fe(III) is reduced to Fe(II) and released from the complex.

*Shewanella* comprise a group of facultative γ-proteobacteria renowned for their respiratory versatility, a feature attributable to a large repertoire of iron-containing proteins, particularly heme-containing proteins such as cytochromes *c* ^5–7^. Because of this*, Shewanella*, as the extensively studied representative *S. oneidensis*, require iron at levels relatively higher than model bacterium *Escherichia coli* ^8^. To synthesize heme, the co-factor of cytochromes and some other proteins such as catalase, *S. oneidensis* utilizes a canonical pathway from glutamate (HemA-H), a highly conserved route for heme biosynthesis in most bacteria^9^. An Fe(II) atom is consumed for each protoheme (heme *b*) at the reaction catalyzed by HemH. The availability of protoheme then allows cytochrome *c* biosynthesis, which is catalyzed by a cytochrome *c* maturation system (Ccm)^10^. In *S. oneidensis*, Fe(II) ions are also consumed by Fe-S proteins, mononuclear non-heme iron and diiron enzymes, albeit in a considerably less amount^11, 12^.

Siderophore-mediated iron uptake by *Shewanella* was first investigated more than 2 decades ago^13^. Putrebactin, the only siderophore produced naturally by *S*. *oneidensis*, is an unsaturated macrocyclic dihydroxamic acid^14–16^. Physiological impacts of this siderophore are uncertain: it was initially reported to be needed for aerobic but not anaerobic growth^17^, but a later work suggested that it plays a role in reduction of iron- and manganese-oxide^17, 18^. In contrast to siderophore, a large number of homologs of the *E. coli* TonB-dependent siderophore receptors are encoded in the *S. oneidensis* genome; among them only SO_2907 has been proposed to play a minor role in iron transport^19^. Moreover, the ABC transporter through which iron-chelator complexes migrate into the cytoplasm remains largely unknown. Iron uptake in *S. oneidensis* may be further complicated by metal reduction, a process that has been a research focus for decades^20^. By exploiting extracellular electron transfer, *S. oneidensis* is able to reduce insoluble Fe(III) species, and importantly, this process could occur under aerobic conditions^21^. Clearly, the mechanism confers cells accessibility to Fe(II). As soluble iron species travel into the periplasm largely freely, the significance of siderophore for iron uptake may be belittled.

Due to the abundance of cytochrome *c*, colonies and cell pellets of *S. oneidensis* are dark-red in general and the color intensity is correlated to cellular cytochrome *c* concentrations^10, 22^. In the course of studies on the cytochrome *c* biosynthesis^10^, we have found by chance, that a spontaneous mutant, SO-X2, develops white colonies on lysogeny broth (LB) agar plates. The phenotype implies that the mutant suffers from abolished or reduced production of cytochrome *c*. However, the mutation was not located in genes for cytochrome *c* biosynthesis, rather, in *putA* (SO_3033), encoding a TonB-dependent ferric siderophore receptor according to the genome annotation. Further analyses confirmed that PutA is responsible for the phenotype and plays a critical role in iron uptake, especially when iron is limited in environments. Furthermore, we found that lactate but not other carbon sources tested can facilitate iron update in a way independent of lactate permeases.

## Methods

### Bacterial strains, plasmids and culture conditions

The bacterial strains and plasmids used in this study are listed in Table 1. Sequences of the primers used in this study are available upon request. All chemicals are from Sigma-Aldrich Co. unless otherwise noted. *E. coli* and *S. oneidensis* were grown aerobically in LB (Difco, Detroit, MI) at 37 and 30 °C for genetic manipulation. When appropriate, the growth medium was supplemented with the following: 2, 6-diaminopimelic acid (DAP), 0.3 mM; ampicillin, 50 μg/ml; kanamycin, 50 μg/ml; gentamycin, 15 μg/ml.

**Table 1:** Strains and plasmids used in this study.

Strain or plasmid	Description	Source or reference
***E. coli*** **strain**		
DH5α	Host strain for plasmids	Lab stock
WM3064	Donor strain for conjugation; Δ*dapA*	W. Metcalf, UIUC
***S. oneidensis*** **strain**		
MR-1	Wild type	Lab stock
SO-X2	A spontaneous mutant from MR-1	This study
HG0265	Δ*ccmI* derived from MR-1	21
HG0266	Δ*ccmF* derived from MR-1	24
HG0827	Δ*lctP1* derived from MR-1	This study
HG1522	Δ*lctP2* derived from MR-1	This study
HG3030	Δ*putA* derived from MR-1	This study
HGLCTP	Δ*lctP1*Δ*lctP2* derived from MR-1	This study
HG0827-3030	Δ*lctP1* Δ*putA* derived from MR-1	This study
HG1522-3030	Δ*lctP2* Δ*putA* derived from MR-1	This study
HGLCTP-3030	Δ*lctP1*Δ*lctP2* Δ*putA* derived from MR-1	This study
HGCYD	Δ*cyd* (Δ*cydABX*) derived from MR-1	42
**Plasmid**		
pHGM01	Ap^r^ Gm^r^ Cm^r^ suicide vector	24
pHG101	Km^r^, promoterless broad-host vector	25
pHG102	pHG101 carrying the *arcA* promoter	25
pHGE-P*tac*	IPTG-inducible P*tac* expression vector	26
pHGEI01	Integrative *lacZ* reporter vector	29
pBBR-Cre	Helper vector for antibiotic marker removal	30
pHG101-0827	Expressing *lctP1* for complementation	This study
pHG101-1522	Expressing *lctP2* for complementation	This study
pHGE-P*tac*-*cyd*	Vector for inducible expression of *cydABX*	42
pHGE-P*tac*-*putA*	Vector for inducible expression of *putA*	This study
pHGEI01-*hemA*	Vector for measuring *hemA* expression	39
pHGEI01-*hemF*	Vector for measuring *hemC* expression	39
pHGEI01-*hemH*	Vector for measuring *ccmA* expression	39
pHGEI01-*putA*	Vector for measuring *putA* expression	This study
pHGEI01-*pub*	Vector for measuring *pubA* expression	This study

For physiological characterization, both LB and defined medium MS supplemented with 30 mM L-lactate as electron donor were used^23^. To test effects of iron species on relevant phenotypes, MS medium contained either Fe(II) [FeSO_4_] or Fe(III) [FeCI_3_] at 3.6 µM. For aerobic growth, overnight cultures of *S. oneidensis* strains were inoculated into fresh medium by 200X dilution, shaken at 200 rpm at 30 °C, and growth was recorded by measuring optical density at 600 nm (OD_600_).

### In-frame mutant construction and complementation

In-frame deletion strains were constructed using the *att*-based fusion PCR method as described previously^24^. In brief, two fragments flanking the genes of interest were amplified by PCR, and then linked by a second round of PCR. The fused fragments were introduced into plasmid pHGM01 using the Gateway BP clonase II enzyme mix (Invitrogen) according to the manufacturer’s instruction. The resulting vectors were maintained in *E. coli* DAP auxotroph WM3064 and subsequently transferred into relevant *S. oneidensis* strains via conjugation. Integration of the deletion constructs into the chromosome was selected by resistance to gentamycin and confirmed by PCR. Verified transconjugants were grown in LB in the absence of NaCl and plated on LB supplemented with 10% sucrose. Gentamycin-sensitive and sucrose-resistant colonies were screened by PCR for intended deletions. Mutants were verified by sequencing the mutated region.

Plasmid pHG101 was used for genetic complementation of the mutants^25^. Wild-type genes and their adjacent promoters, were generated by PCR, cloned into pHG101, and the resultant vectors were transferred into relevant *S. oneidensis* strains by conjugation via *E. coli* WM3064. For inducible gene expression, genes of interest generated by PCR were placed under the control of Isopropyl β-D-1-thiogalactoside (IPTG)-inducible promoter P*tac* within pHGE-P*tac* ^26^. After verification by sequencing, the resultant vectors were transferred into the relevant strains via conjugation.

### Identification of genes capable of suppressing the phenotype of SO-X2

*S. oneidensis* genomic DNA partially digested with the restriction enzyme *Sau*3AI was separated by agarose gel electrophoresis. DNA fragments of 1–8 kb were recovered from the gels and ligated to *Bam*HI-digested vector pHG102, which carries a relatively constitutive promoter for the *S. oneidensis arcA* gene^23, 26^. Ligated plasmid DNA was introduced into *E. coli* WM3064 by electroporation; approximately 4 × 10^5^ independent colonies were pooled, dubbed as the *S. oneidensis* genomic library, which was aliquotted for immediate use and stored at −80 °C.

*S. oneidensis* mutant SO-X2 was conjugated with WM3064 carrying the *S. oneidensis* genomic library on LB plates and the transconjugants were plated onto the selective medium. From multiple attempts, a total of 7 colonies that harbor potential suppressing genes were obtained, from which plasmids were extracted and sequenced.

### Culture color analysis

The analysis was performed both on agar plates and in liquid medium. For agar plates, overnight cultures were streaked for obtaining discrete colonies. Alternatively, overnight cultures were adjusted to an OD_600_ of ∼1, followed by 10-fold serial dilutions, and 5 µl of each dilution was spotted onto agar plates. The plates were incubated at 30 °C before being read. In liquid medium, cultures grown to the mid-log phase (OD_600_ of ∼0.3, the same afterwards) were centrifuged at 4000 rpm for 10 min and pellets were photographed.

### Determination of heme *c* levels

Cells of the mid-log phase were harvested and then were lysed with lysis buffer (0.25 M Tris/HCl, (pH 7.5), 0.5% Trion-X100). In this study, protein concentration was determined with a bicinchoninic acid assay kit with bovine serum albumin (BSA) as a standard according to the manufacturer’s instructions (Pierce Chemical). The amount of heme *c* was measured following the procedure described elsewhere^27^.

Abundance of cytochromes *c* was also estimated by heme staining. Cells of the mid-log phase were harvested, washed with phosphate buffered saline (PBS), resuspended in the same buffer, and sonicated. The cell lysates were resolved by sodium dodecyl sulfate polyacrylamide gel electropheresis (SDS-PAGE) using 12% gels and stained with either Coomassie brilliant blue or 3,3′,5,5′-tetramethylbenzidine as described elsewhere^28^.

### Analysis of gene expression

The activity of promoters was assessed using a single-copy integrative *lacZ* reporter system as described previously^29^. A fragment containing the sequence upstream of operons under test from −300 to +1 (relative to the translation start codon) was amplified and cloned into the reporter vector pHGEI01 and verified by sequencing. The resultant vector was then transferred by conjugation into relevant *S. oneidensis* strains, in which it integrated into the chromosome and the antibiotic marker was then removed by an established approach^30^. Cells grown to the mid-log phase under conditions specified in the text and/or figure legends were collected and β-galactosidase activity was determined by monitoring color development at 420 nm using a Synergy 2 Pro200 Multi-Detection Microplate Reader (Tecan) presented as Miller units^29^.

For qRT-PCR, cells of the mid-log phase were harvested by centrifugation and total RNA was isolated using RNeasy Mini Kit (QIAGEN) according to the manufacturer’s instructions. The analysis was carried out with an ABI7300 96-well qRT-PCR system (Applied Biosystems) as described previously^31^. The expression of each gene was determined from three replicas in a single real-time qRT-PCR experiment. The Cycle threshold (*C*_*T*_) values for each gene of interest were averaged and normalized against the *C*_*T*_ value of the *arcA* gene, whose abundance is relatively constant during the log phase. Relative abundance (RA) of each gene was presented.

### Quantification of intracellular total iron

Quantification of total iron was carried out with the established method^32^. Cells grown overnight on LB plates or to the mid-log phase in LB were collected, washed with PBS, and adjust to similar densities (∼0.6 of OD_600_). Aliquots of 50 ml were mixed with 5 ml of 50 mM NaOH and sonicated on ice, and centrifuged at 5000 *g* for 10 min. The cell lysates (100 μl) were then mixed with 100 μl 10 mM HCl and 100 μl iron releasing reagent (a freshly mixed solution of equal volumes of 1.4 M HCl and 4.5% (w/v) KMnO_4_) and treated at 60 °C for 2 hours. After cooling, the iron detection reagents (6.5 mM ferrozine, 6.5 mM neocuproine, 2.5 M ammonium acetate, and 1 M ascorbic acid in water) were added. The absorbance of samples was measured at 550 nm 30 min later. The standard curve was depicted using FeCI_3_ up to 300 μM.

### Siderophore assays

In order to assess putrebactin production and secretion, *S. oneidensis* strains were grown in LB, MS, and iron-limited MS (1 µM FeCl_3_) to the stationary phase and cell-free culture supernatants were obtained by centrifugation. Siderophore within the supernatants was quantified using the Chrome Azurol S (CAS) assay^33^. For visualization, *S. oneidensis* strains grown on LB, MS, and iron-limited MS agar plates were subjected to direct detection of siderophore^33^.

### Nitrite susceptibility assay

*S. oneidensis* strains grown to the mid-log phase were adjusted to approximately 10^8^ colony forming units (CFU)/ml, and followed by 10-fold serial dilutions. Five µl of each dilution was spotted onto LB plates containing 5 mM nitrite (NaNO_2_). The plates were incubated at 30 °C before being read.

### Nadi assay

Visual analysis of the *cbb*_3_-HCO activity was done by staining colonies with the agents for the Nadi Assay. Nadi reactions were carried out by the addition of α-naphthol and *N’,N’*-dimethyl-p-phenylenediamine (DMPD) on LB agar plates^34^. Colonies were timed for formation of the indophenol blue.

### Other analyses

Homologues of proteins of interest were identified via a BLASTp search of the NCBI’s nonredundant protein database, using the amino acid sequence as the query. Student’s *t* test was performed for pairwise comparisons. Values were presented as means ± standard error of the mean (SEM).

## Results

### Characteristics of *S. oneidensis* spontaneous mutant SO-X2

**Figure 1:**
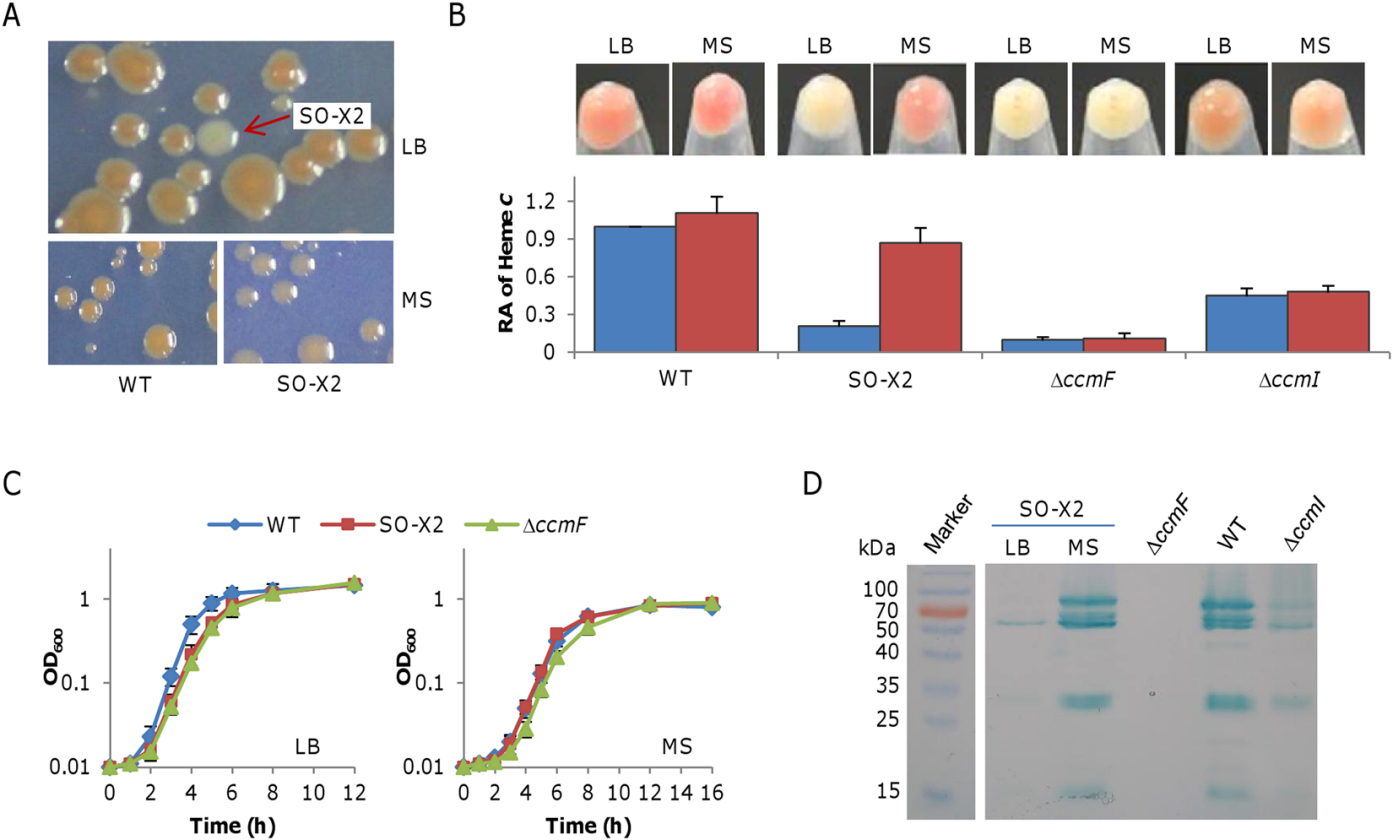
Characteristics of spontaneous mutant SO-X2. (**A)** Colonies on LB and MS agar plates. On MS plates, the wild-type (WT) and SO-X2 from the LB plate were cultivated independently. **(B)** Heme *c* levels in SO-X2. Cultures (∼0.6 of OD_600_) of indicated strains were pelletted and photographed, then were lysed for quantition of heme *c* levels. The data were first adjusted according to protein levels of samples, and then the averaged levels of the mutants was normalized to that in the wild-type, which was set to 1, giving to relative abundance (RA). ∆*ccmF* and ∆*ccmI*, which completely and partially lose capabilities of producing cytochromes *c* respectively, were included for comparison. Note that cultures at all growing phases (log and stationary) in LB and MS showed similar results. **(C)** Growth of SO-X2 in LB and MS. Fresh media were inoculated with overnight cultures to ∼0.01 of OD_600_, and incubated (200 rpm) under aerobic conditions. **(D**) Heme staining. Proteins (10 μg per lane) extracted from the indicated samples were resolved by SDS-PAGE and analyzed by heme staining. All experiments were performed at least three times and presented either as means ± SEM or by a representative of similar results.

This investigation began with the chance observation that an *S. oneidensis* spontaneous mutant SO-X2 lost the feature, developing white color (WC phenotype) colonies on LB agar plates (Fig. 1A). However, when we made an attempt to characterize SO-X2 on defined medium MS agar plates, colonies were red-colored (RC phenotype) (Fig. 1A). Similar observations were obtained from liquid MS, as evidenced by cell pellets from cultures of different growing phases (Fig. 1B). These data suggest that the WC phenotype of SO-X2 is conditional, associated with certain agents that may be medium ingredients and/or metabolic intermediates in cultures. Additionally, compared to the wild-type, SO-X2 was modestly defective in growth in liquid LB but not in liquid MS (Fig. 1C). Thus, the cell color and growth appear to be linked.

An analysis of intracellular heme *c* levels in SO-X2 confirmed that the WC phenotype is indeed due to the substantially reduced production of cytochrome *c* whereas in cells grown in MS medium these proteins were more abundant, nearly comparable to the wild-type (Fig. 1B). This was in contrast to a *ccmF* (SO_0266, encoding cytochrome *c* maturation protein lyase CcmF) mutant, which is unable to produce any cytochrome *c*, resulting in WC phenotype in both media. The difference implies that the cytochrome *c* maturation system in SO-X2 may not be damaged. With respect to growth, we found that Δ*ccmF* was not distinguishable from SO-X2 when grown in LB, but unlike SO-X2, Δ*ccmF* carried a defect in MS (Fig. 1C). In *S. oneidensis*, oxygen respiration is carried out efficiently by cytochromes *c*, including *bc*_1_ complex and *cbb*_3_ oxidase and the Δ*ccmF* strain was previously shown to have a growth defect^24, 29, 35^. Thus, these data support that the growth defect of SO-X2 is linked to its cytochrome *c* levels.

For further confirmation, we used Δ*ccmI*(SO_0265), in which cytochrome *c* production is compromised but not abolished^10^. When grown in both LB and MS media, Δ*ccmI* exhibited similar color and had a cytochrome *c* content about 45% relative to that of the wild-type (Fig. 1B), indicating that impairments in the Ccm system are unconditional to cytochrome *c* biosynthesis. Hence, the phenotype of SO-X2 is not due to an impaired Ccm system. This was further validated by heme-staining (Fig. 1D). The assay revealed that WC cells of SO-X2 produced cytochromes *c* at substantially reduced levels whereas RC cells from MS had cytochromes *c* nearly as abundant as the wild-type.

### Identification of the mutation in SO-X2

To map the mutation, we constructed an *S. oneidensis* genomic library on expression vector pHG102 driven by the *S. oneidensis arcA* promoter, whose activity is relatively constitutive^22^. The library was introduced into SO-X2, and after multiple attempts on LB agar plates 7 red colonies comparable to those of the wild-type were obtained, namely SO-X2^S^ for suppressor strains. Sequencing vectors extracted from SO-X2^S^ revealed that all of them harbored *putA*(SO_3033), which encodes a TonB-dependent ferric putrebactin siderophore receptor of 730 amino acids.

**Figure 2:**
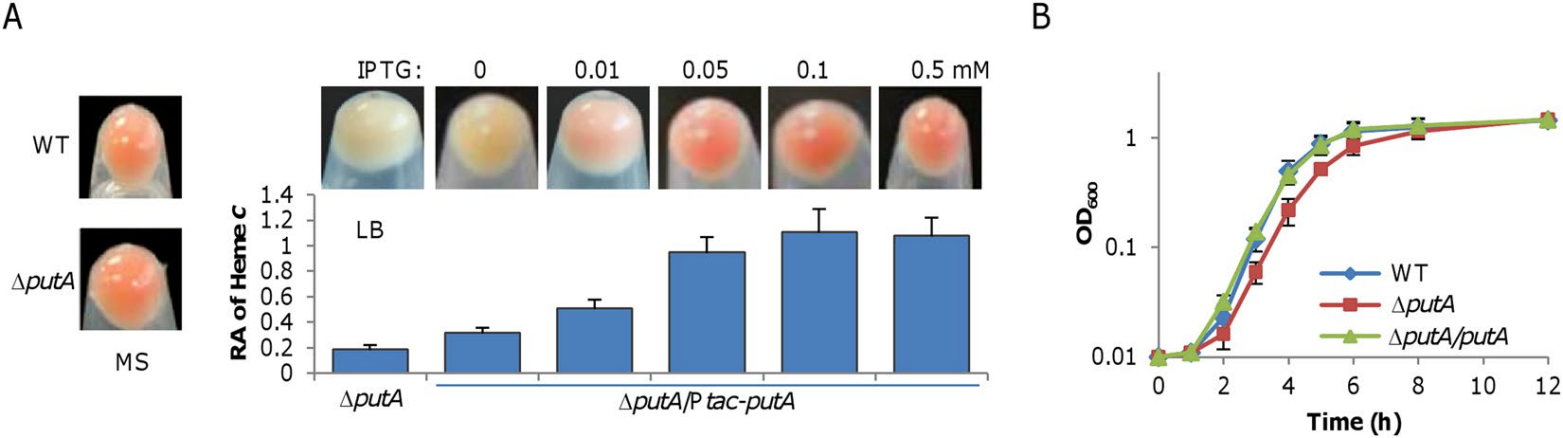
The *putA* gene underlies phenotypes of SO-X2. (**A)** The culture color phenotype and heme *c* levels. Complementation of the *putA* deletion mutant was performed with IPTG-inducible promoter P*tac* driving *S. oneidensis putA*. Assays were performed the same as in Fig. 1B. (**B**) Growth of the *putA* mutant in LB. IPTG concentration, 0.5 mM. All other strains carry empty vector. Experiments were performed at least three times and presented either as means ± SEM or by a representative of similar results.

For confirmation, the *putA* region of SO-X2 was cloned and sequenced. There was a transversion of T43A, resulting in a nonsense mutation (**A**GA → **T**GA), which leads to an incomplete polypeptide. Given that the mutation is near the beginning of the gene, it is definite that the truncated polypeptide is inactive. Thus, these data suggest that the WC phenotype of SO-X2 grown in LB is likely due to the loss of PutA. A *putA* in-frame deletion strain was then constructed; with respect to all characteristics of SO-X2 revealed above, this ∆*putA* strain was indistinguishable (Fig. 2A and B). We then performed genetic complementation of the ∆*putA* strain by expressing a copy of *putA* from IPTG-inducible promoter P*tac*, whose activity increases linearly with IPTG up to 0.5 mM^26, 36, 37^. As shown in Fig. 2A, a perfect restoration of color and heme *c* level was achieved with 0.05 mM IPTG and excessive PutA, for example, induced by 0.5 mM IPTG, at least 10x over 0.05 mM according to previous calibration^36^, had no beneficial or detrimental effect. Notably, a slightly improved production of cytochromes *c* in the absence of IPTG was observed, a scenario reported before because of the leakiness of the promoter^36, 37^. In parallel, growth defect was perfectly corrected with IPTG at 0.5 mM (Fig. 2B). These data, all together, conclude that the *putA* mutation accounts for the observed phenotypes of SO-X2.

### PutA is critical for iron import under iron-limit conditions

**Figure 3:**
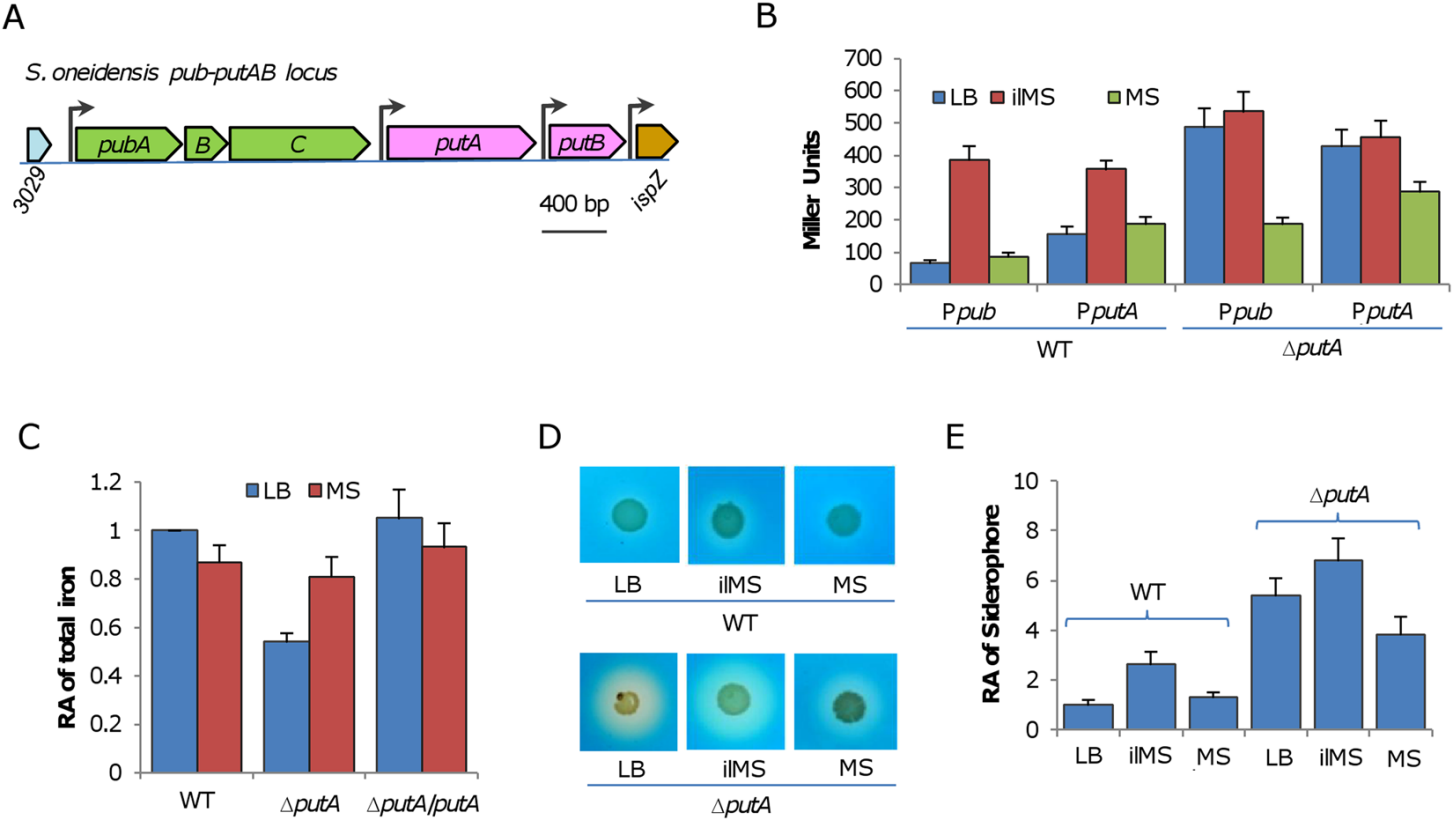
Physiological impacts of PutA in *S. oneidensis*. (**A)** Gene organization of the *putA* locus in *S. oneidensis*. The locus is largely conserved in *Shewanella*. **(B)** Expression of indicated operons in the wild-type and ∆*putA* analyzed by an integrative *lacZ*-reporter. Cells of the mid-log phase were prepared as described in Methods, in which activity of promoters for *pub* and *putA* operons, P*pub* and P*putA* respectively, was assayed. ilMS, iron-limited MS. **(C)** Total iron in the wild-type and ∆*putA*. Cultures (∼0.6 of OD_600_) of indicated strains were pelletted and subjected to the assay. The data were first adjusted according to protein levels of samples, and then the averaged levels of the mutants was normalized to that in the wild-type, which was set to 1, giving to relative abundance (RA). **(D)** Siderophore production. Wild-type and the *putA* mutant were grown on LB, MS, and iron-limited MS (ilMS) agar plates for 24 hours. Siderophore was examined by the CAS assay. **(E)** Quantification of siderophore production. Wild-type and the *putA* mutant were grown in LB, MS, and iron-limited MS (ilMS) to the stationary phase, and the CAS activities of supernatants were examined. The CAS values were first adjusted according to protein levels of samples, and then the averaged levels of the mutants was normalized to that in the wild-type, which was set to 1, giving to relative concentration (RC). All experiments were performed at least three times and presented either as means ± SEM or by a representative of similar results.

The *putA* gene is located in the *pubABC*-*putA*-*putB*(SO_3030-4) cluster (Fig. 3A), which is largely conserved in Shewanellae (∼70% of the sequenced). While PubABC are enzymes responsible for putrebactin siderophore production, PutA and PutB are predicted to be involved in the uptake and utilization of ferric-putrebactin^15^. However, PutB, annotated as ferric-siderophore reductase, has no detectable role in physiology^17^, casting doubt on the role of PutA in siderophore-dependent iron uptake. Moreover, TonB-dependent receptors are many, including 7 siderophore receptors (Table S1); In fact, given comparable sequence similarities among these proteins, any such protein may function in iron uptake^19^.

Production of siderophore is induced under low-iron conditions^2^. If PutA is the receptor for putrebactin, we reasoned that the *putA* gene would be responsive to iron levels as the *pubABC* operon. By using an integrative *lacZ* reporter, we found that the promoter of the *pub* operon was low in activity in cells grown in both LB and MS, but showed substantially enhanced activity in cells grown in iron-limited MS (ilMS, containing 1 µM FeCl_3_) (Fig. 3B). A similar trend was observed from the *putA* promoter although induction was less drastic, supporting that PutA is associated with PubABC in functionality.

In parallel, if PutA is the receptor for putrebactin, the ∆*putA* strain grown in LB should suffer from an iron shortage. To test this, we measured total iron in ∆*putA* WC and RC cultures. Wild-type cells, used as the control, had total iron at 5.12 and 4.45 nmol/mg protein for cultures grown in LB and MS, respectively (Fig. 3C). In ∆*putA* WC cells grown in LB, there was substantial decrease in intracellular total iron concentrations, approximately 54% relative to the wild-type, data in excellent agreement with the results of a previous study^18^. In contrast, iron levels in ∆*putA* RC cells and the wild-type grown in MS were comparable (Fig. 3C). These data manifest that cells of the ∆*putA* WC culture, but not of the RC culture, are indeed low in iron content.

Given low iron levels in ∆*putA* WC cells, it is expectable that siderophore production in ∆*putA* would be enhanced. Indeed, siderophore levels in cultures of the wild-type and ∆*putA* strains grown on LB differed significantly, represented by halos of ∼1 and 7 mm in radius respectively (Fig. 3D). When grown in MS medium, the difference became much smaller, largely due to reduced siderophore production in the ∆*putA* strain. Furthermore, in ilMS medium, in which ∆*putA* grew into white culture, both the wild-type and ∆*putA* increased putrebactin production substantially. In parallel, we quantified siderophore levels in supernatants obtained from the wild-type and ∆*putA* strains grown in these media. As shown in Fig. 3E, the data were in excellent agreement with those presented in Fig. 3D. The increase in siderophore production resulting from the *putA* mutation was reduced to the wild-type level when a copy of the *putA* gene was expressed *in trans* (Fig. S1). In addition, we examined expression of the *pub* operon, as well as *putA*, in ∆*putA* and found that the result was correlated well with their iron levels (Fig. 3B). These data strongly suggest that PutA is a putrebactin siderophore acceptor, which is particularly important in iron uptake from iron-limit environments.

### Iron dictates biosynthesis of heme *b*

**Figure 4:**
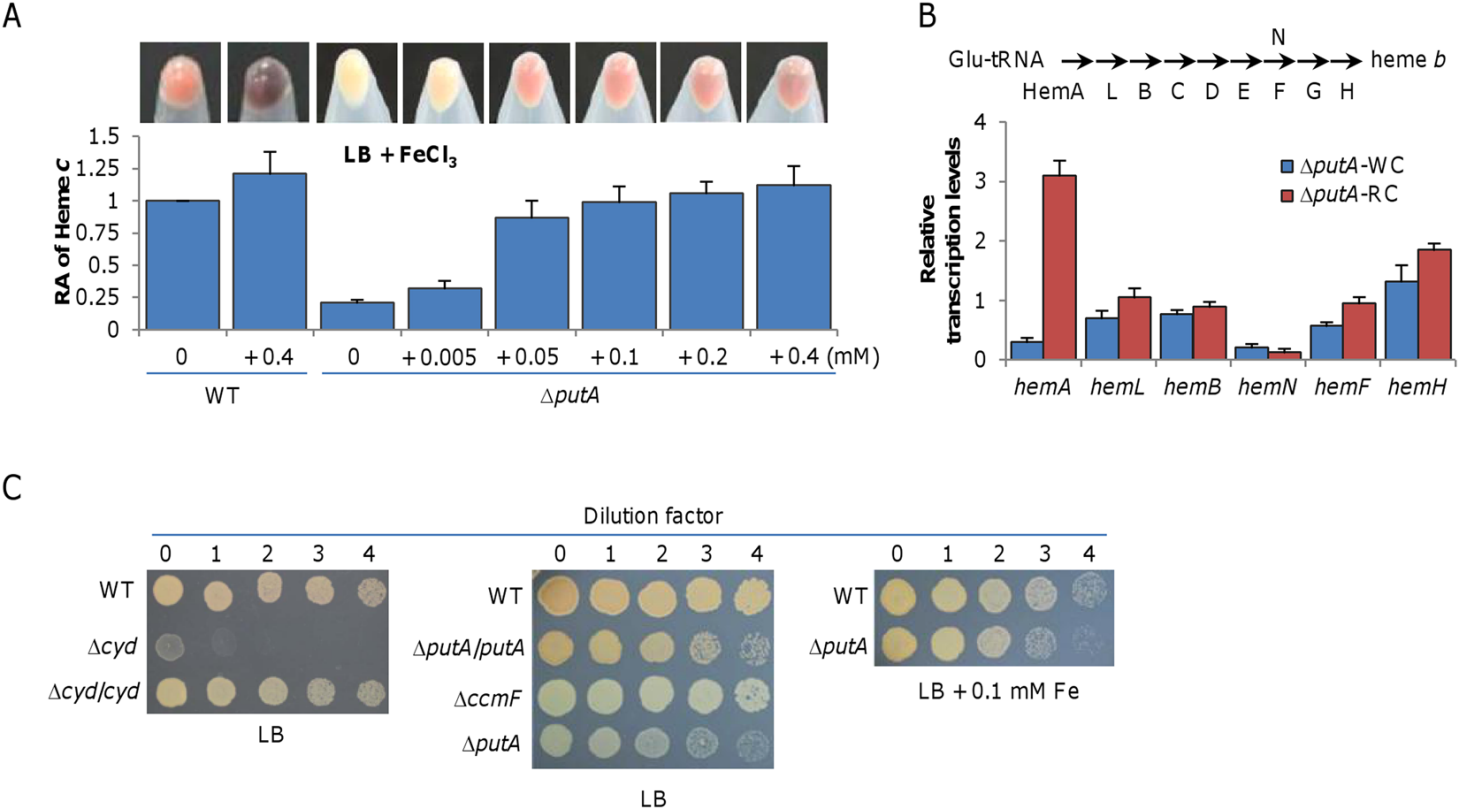
Iron can complement the defect of the putA mutant in production of cytochromes *c*. (**A)** Iron influences heme *c* levels in ∆*putA*. Cultures (∼0.6 of OD_600_) of WT and ∆*putA* grown in LB with iron at varying concentrations were pelletted and photographed, then were lysed for quantition of heme *c* levels as above. Note that Fe(III) was reduced extracellularly to form Fe_3_O_4_ particles in both WT and ∆*putA* when its concentrations were high. **(B)** Expression of the *hem* genes in ∆*putA* analyzed by qRT-PCR. Enzymes for heme biosynthesis are shown above. Cells of mid-log phase grown in LB (WC) and MS (RC) were prepared as described in Methods. The averaged expression level of each gene in mutants was normalized to that of the *arcA* gene, which is relatively constant. **(C)** Nitrite susceptibility of ∆*putA*. Nitrite susceptibility of *S. oneidensis* is dictated by cytochrome *bd* oxidase. Cells at 10^8^ cfu/ml were serial diluted and 5 µl of each dilution was dropped on LB plates containing 5 mM nitrite. All experiments were performed at least three times and presented either as means ± SEM or by a representative of similar results.

The WC phenotype of the ∆*putA* strain in LB coincides with low iron contents in cells, suggesting a link between iron levels and the cytochrome *c* content. To test this, we grew the ∆*putA* strain in LB supplemented with iron of varying concentrations. Color of cell pellets turned from whitish to reddish with iron (Fig. 4A). When iron reached 0.05 mM, the ∆*putA* strain had pellet color resembling that of the wild-type grown in LB. Notably, excessive iron in cultures containing 0.4 mM FeCl_3_ was reduced to Fe_3_O_4_ particles by both wild-type and ∆*putA* strains, but the reducing rate in the former was significantly faster than that in the latter. The heme *c* quantification confirmed that the red-color ∆*putA* culture has a comparable amount of cytochromes *c* (Fig. 4A). These data indicate that the ∆*putA* strain is normal in iron uptake when iron is abundant, further supporting that PutA works in iron-limited conditions, a scenario that is consistent with siderophore function. In addition, the data also conclude that iron scarcity in the ∆*putA* WC culture underlies deficiency in cytochrome *c* synthesis.

To elucidate that iron is the predominant factor for decreased production of cytochromes *c* in ∆*putA* WC cells, we focused on the heme synthetic pathway, which consumes iron^38^. As protoporphyrin and other heme precursors can be toxic, heme biosynthesis is believed to be coordinated with iron availability. *S. oneidensis* possesses the most common pathway for heme synthesis (Fig. 4B), which entails nine reactions that converts glutamyl-tRNA to protoporphyrin IX^39^. Genes in heme synthesis are conditionally inducible include *hemA*(SO_3834), *hemL*(SO_1300), *hemB*(SO_2587), *hemN*(SO_4730), *hemF*(SO_0038), and *hemH*(SO_2019)^38, 40, 41^. To explore which steps are affected in ∆*putA* WC cells, we monitored abundance of the transcript of these *hem* genes by qRT-PCR. In ∆*putA* WC and RC cells, the *hemA* gene was transcribed at drastically different levels; the transcript in RC cells was 10-fold more abundant than that in WC cells, a result in line with culture color and heme levels. In contrast, the difference in transcription of the remaining genes between these two types of cells was at most modest, if not insignificantly (Fig. 4B). To confirm this observation, we used the *lacZ*-reporter to assay β-galactosidase activities driven by *hemA*, *hemF*, and *hemH* promoters and results were similar (Fig. S2). Given that the reaction carried out by HemA is the rate-limiting step^38^, these data imply that iron limitation down-regulates the entire pathway, rather than specific steps.

Oxygen respiration is carried out by cytochrome *cbb*_3_ and *bd* oxidases in *S. oneidensis*, of which the former is the only enzyme reacting with the Nadi reagents in *S. oneidensis* ^35^. As two subunits (CcoP(SO_2361) and CcoO(SO_2363)) of the *cbb*_3_ oxidase are cytochromes *c*, it is expected that activity of the *cbb*_3_ oxidase would decrease significantly, if not lose completely. To test this, the Nadi plate assay was performed. While the wild-type colonies generated a blue ring in 2 min, ∆*putA* WC colonies, resembling those of a *cbb*_3_-HCO-deficient strain (∆*cco*(SO_2361-4)), could not generate a faint blue coloration in an incubation of 5 min (Fig. S3). Hence, ∆*putA* WC cells lose production of the *cbb*_3_ oxidase. Although heme synthesis is down-regulated, protoheme IX (heme *b*) must be made in ∆*putA* WC cells because heme is essential to aerobiosis of *S. oneidensis* ^35^. In the absence of cytochrome *c*, cytochrome *bd* alone can support growth, albeit less efficiently. To assess impacts of PutA on biosynthesis of heme *b*, we assayed resistance to nitrite, which is proportional to cytochrome *bd* activity^30^. As shown in (Fig. 4C), the nitrite resistance of the ∆*putA* strain either in WC or RC form was comparable to that of the wild-type, but substantially higher than that of the *cyd* mutant (∆*cyd*(SO_3284-6)), a previously verified strain lacking the *bd* oxidase^42^. Thus, ∆*putA* WC and RC cells have sufficient amounts of the *bd* oxidase despite overall reduced production of protoheme IX.

### Lactate facilitates iron import in *S. oneidensis*

**Figure 5:**
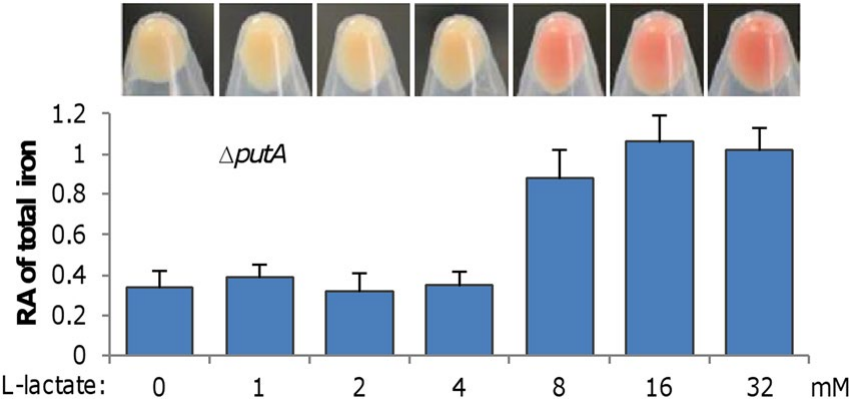
Lactate facilitates iron uptake in ∆*putA*. Cultures (∼0.6 of OD_600_) of ∆*putA* grown in LB with L-lactate at varying concentrations were pelletted and photographed, then were lysed for quantition of iron levels. The data were first adjusted according to protein levels of samples, and then the averaged levels of the mutant was normalized to that in the wild-type, which was set to 1, giving to relative abundance (RA). The experiments were performed at least three times and presented either as means ± SEM or by a representative of similar results.

Although iron levels explain why Δ*putA* forms red colonies on LB plates with iron, it does not appear to be the reason for Δ*putA* to display RC phenotype when grown in MS medium because MS contains 3.6 µM iron, much lower than LB (17 µM iron)^43^. To unravel the regulatory mechanism underlying the increased iron uptake under this condition, we first tried to figure out whether certain ingredients of the MS medium stimulate the process. As the carbon source in MS, L-lactate, is only ingredient that differs substantially in amount between two media, effects of L-lactate addition on the culture color of Δ*putA* grown in LB were examined. L-lactate was added into LB to final concentrations ranging from 0 to 32 mM, increased by 2-fold. The Δ*putA* culture remained white in the presence of no more than 4 mM L-lactate, but further increase to 8 mM and higher turned the culture to red (Fig. 5). An analysis of iron concentrations in these samples revealed that the RC cells have increased iron levels, suggesting that L-lactate may be the decisive agent of MS that promotes iron uptake of Δ*putA*.

*S. oneidensis* can metabolize both L- and D-lactate^44^, but there is a difference in utilization rates of these two isomers^45^. However, in LB supplemented with D-lactate, a similar effect was observed (Fig. S2), indicating that the iron-uptake promotion of lactate is irrespective of isomerism. We then assessed effects of other carbon sources in MS and LB, including acetate, pyruvate, and N-acetylglucosamine(NAG), all of which are good electron donors for supporting growth^45, 46^. Clearly, none of these was able to make the Δ*putA* culture reddish (Fig. S4), indicating that the capability of transporting iron may be restricted to lactate.

### Iron-uptake mediated by lactate does not depend on lactate permeases

To address why lactate can facilitate iron uptake, we focused on the route through which lactate is imported. In bacteria, acquisition of lactate relies on lactate permease, which generally is capable of transporting both L- and D-lactate isomers^47^. In *S. oneidensis*, genes that encode enzymes metabolizing lactate and their regulatory proteins have been studied^44, 45^, but those for lactate transport remain untouched. The *S. oneidensis* genome encodes two putative lactate transporters, SO_1522 and SO_0827; the former is clustered with metabolizing genes (*dld*(SO_1521), D-lactate dehydrogenase; *lldEFG*(SO_1518-20), L-lactate dehydrogenase) for both D- and L-lactate, while the latter is located somewhere on the chromosome. However, a sequence comparison analysis revealed that SO_0827 is likely to be a genuine lactate permease based on its E-values of BLASTp to both *E. coli* lactate permeases, LldP and GlcA, which are 0 (Table S2). In contrast, whether SO_1522 can function as a lactate permease is not certain as its sequence similarities to *E. coli* lactate permeases are rather modest.

**Figure 6:**
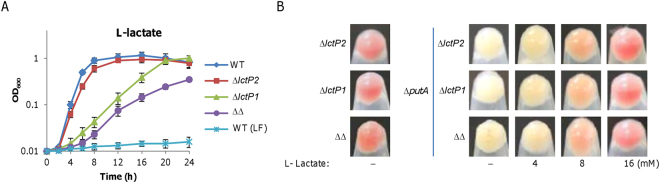
Iron uptake mediated by lactate is independent of lactate permeases. (**A**) Effect of lactate permease loss on growth on lactate. Deletion strains for *lctP1*(SO_0827), *lctP2*(SO_1522), and both (∆∆) were grown in MS with 30 mM lactate as carbon source. For all strains under test, no growth was observed in lactate-free (LF) MS; only WT was shown. **(B)** Effect of lactate permease loss on culture color of ∆*putA*. Cultures (∼0.6 of OD_600_) of indicated strains grown in LB with L-lactate at varying concentrations were pelletted and photographed. All experiments were performed at least three times and presented either as means ± SEM or by a representative of similar results.

To test roles of SO_0827 and SO_1522 in lactate uptake, we constructed mutants lacking each or both of these genes. In MS medium containing 30 mM Lactate, loss of either SO_0827 or SO_1522 led to impaired growth (Fig. 6A), indicating that both systems function as lactate permeases. Clearly, the SO_1522 deletion impacted growth more severely than the SO_0827 depletion, suggesting that SO_1522 is the predominant system for lactate import. Interestingly, the ∆SO_0827∆SO_1522 strain was able to grow, albeit at further reduced rates. As this growth was significant compared to that in the absence of lactate, it is apparent that *S. oneidensis* has backup routes for lactate uptake. These observations were validated by genetic complementation; expression of each of the missing genes *in trans* restored the corresponding phenotype (Fig. S5A). Moreover, similar results were obtained with D-lactate (Fig. S5B). Thus, both SO_0827 and SO_1522 are authentic LD-lactate permeases in *S. oneidensis*, and because of this, we named them LctP1 and LctP2, respectively.

In LB supplemented with 30 mM L-lactate, none of ∆*lctP1*, ∆*lctP2*, and ∆*lctP1*∆*lctP2* was distinguishable from the wild-type with respect to growth rate (Fig. S5C), implicating that impacts of these mutations are insignificant when grown in media that suffice for the optimal growth. Additionally, cultures of these mutants grown in LB were all red (Fig. 6B), indicating that the contribution of lactate permeases in iron uptake is negligible in the wild-type background. To assess the involvement of lactate permeases in iron uptake, we removed *lctP1*, *lctP2*, or both from the *putA* mutant. The resulting mutants, ∆*putA*∆*lctP1*, ∆*putA*∆*lctP2*, and ∆*putA*∆*lctP1*∆*lctP2*, were examined in LB supplemented with L-lactate of varying concentrations up to 30 mM. Compared to the ∆*put* strain, all three mutants grew similarly in LB without or with L-lactate (Fig. S5D). In the case of culture color, they also resembled their parental strain by turning into reddish with lactate of 8 mM or more (Fig. 6B). These data indicate that iron-transport mediated by lactate is independent of lactate permeases in *S. oneidensis*.

## Discussion

**Figure 7:**
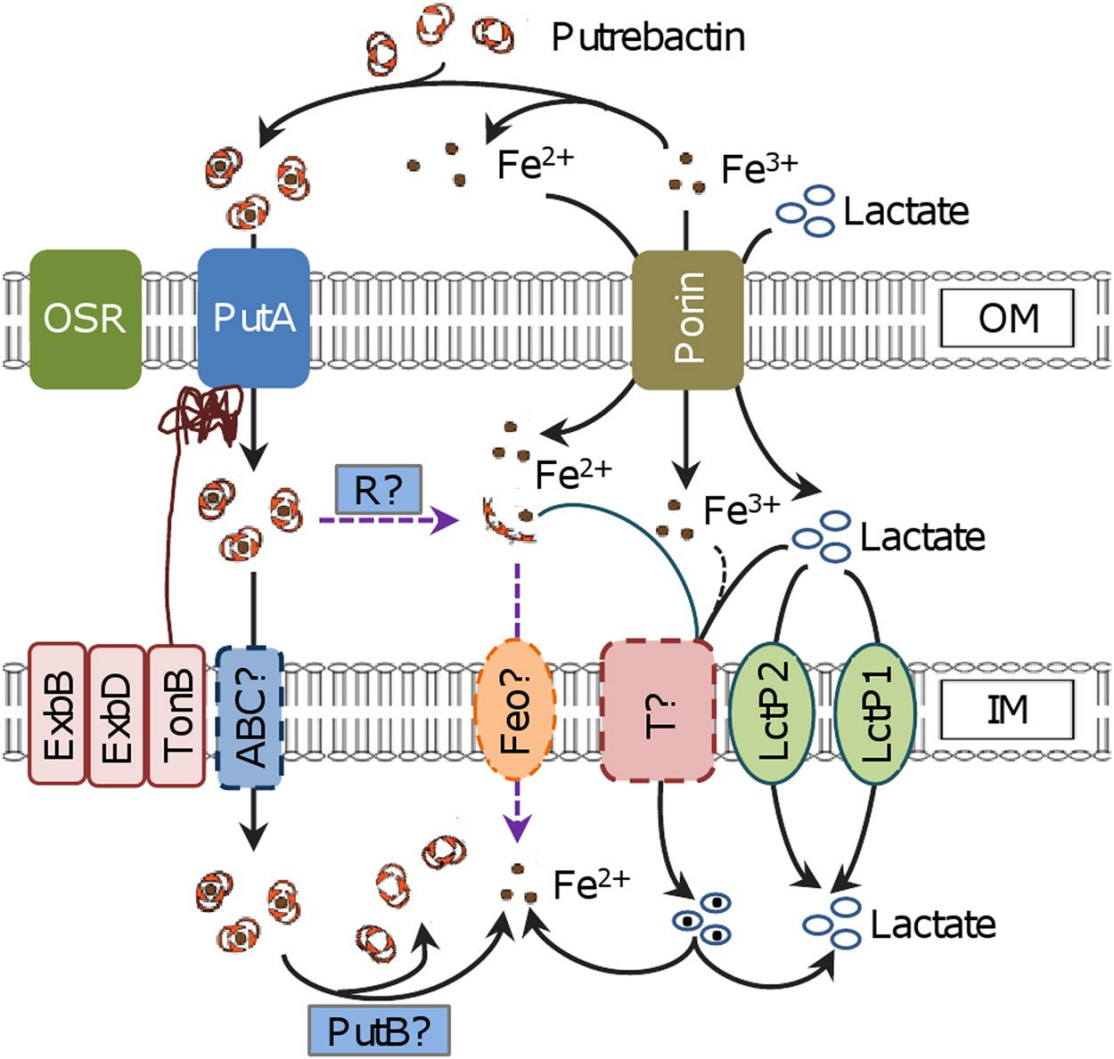
Model for iron uptake in *S. oneidensis*. Fe(III) in the environment can be reduced to Fe(II) and scavenged by putrebactin produced and secreted by *S. oneidensis* to form Fe(III)-putrebactin complexes. The complex enters the periplasm through TonB-dependent PutA and other siderophore receptors (OSR) are probably not involved in the process. Either through unknown ABC transporter (ABC?) or reductase (R?), the complex is imported into the cytoplasm or releases Fe(II), respectively. Fe(II) may be imported by Feo transporter (Feo?) across IM or by lactate through unknown transporter (T?). Lactate enters the cytoplasm mainly through lactate permeases LctP2 and LctP1.

*Shewanella* thrive in redox-stratified environments because of their respiratory versatility, largely based on a large repertoire of iron-containing proteins^6^. Naturally, it is found that *S. oneidensis* has high concentrations of iron relative to *E. coli* ^8^. As a consequence, the organism may require robust systems for iron uptake. In this study, we have performed a genetic analysis of iron uptake in *S. oneidensis*, providing insights into the role of siderophore receptor PutA with respect to its functionality. As shown in the conceptual model (Fig. 7), PutA is a key factor for iron uptake under low iron conditions; its loss results in an iron shortage, which in turn compromises heme and cytochrome *c* synthesis, leading to the white-color phenotype. Furthermore, we presented data to indicate that lactate promotes iron uptake in a way independent of lactate permeases.

Although PubABC of *Shewanella* can synthesize several macrocyclic dihydroxamic acid siderophores with addition of specific precursors, putrebactin is the only siderophore produced naturally and its physiological impacts have been in dispute^16–18^. In contrast to siderophore, TonB-dependent receptors are rather abundant. To date, SO_2907 is the only one that has been demonstrated to be involved in iron transport, although its significance is marginal^19^. Several lines of evidence, particularly those presented in this study, support that PutA (SO_3033) is a specific TonB-dependent receptor for putrebactin. The *putA* and *pubABC* genes, clustered on the chromosome, are responsive to iron in a coordinating way: induced by iron limitation. In the absence of PutA, *S. oneidensis* is severely defective in iron uptake when iron is limited, a condition that best fits the function of siderophore. While the ABC transporter responsible for iron-siderophore across IM remains to be defined, the annotated ferric putrebactin reductase PutB has been previously shown to be dispensable for iron physiology^17^, implying that iron-siderophore reduction may be carried out by other proteins. We are working to identify them.

Given the abundance of TonB-dependent receptors in *S. oneidensis*, we anticipated that some of them may make a difference in iron uptake in the absence of PutA (Fig. 7). We were surprised when that turned out not to be the case. One of possibilities is that the receptor for putrebactin has to be highly specific; other receptors could not recognize the siderophore despite considerable sequence similarities. Perhaps some of these receptors can work with other siderophores released from microorganisms coexisting with *Shewanella* in the same environment, a scenario that has been widely reported in many bacterial species^48^. Iron-siderophore is also possibly to be degraded first in the periplasm, during which Fe(III) is reduced to Fe(II) by an unknown reductase (Fig. 7). Subsequent uptake of Fe(II) is probably mediated by ferrous iron transporter FeoAB(SO_1873-4), a ubiquitous bacterial iron uptake system^49^. However, this merits further investigation.

Lactate is used as a carbon and energy source for the majority of studies involving *Shewanella* spp. grown in defined medium under both oxic and anoxic conditions^44, 45^. In this study, we found that in *S. oneidensis* this chemical is able to facilitate iron uptake. Lactate can chelate both Fe(II) and Fe(III), forming distinct chelates: ferrous lactate is composed of two lactate molecules and one iron whereas ferric lactate has a ratio of 1 to 1; the stability constant of lactate-Fe(III) is at least two degrees higher than that of lactate-Fe(II)^50, 51^. In addition, effects of lactate on transport of Fe(II) and Fe(III) across the cell membrane are opposite in eukaryotic cells, where lactate inhibits Fe(II) but promotes Fe(III) transport^51, 52^. It should be noted that lactate-iron(III) chelates, whose stability constant is 6.4, is substantially inferior to siderophores for iron scavenging, whose stability constants are usually over 30^53^. Hence, lactate plays an insignificant role in iron uptake when a siderophore-based route is functioning. In the absence of such routes, however, the contribution by lactate in iron uptake apparently turns to be critical to *Shewanella*.

We do not yet know the route of the lactate-based iron transport (Fig. 7). Given that the loss of both lactate permeases does not block the process, it is clear that the route is independent of the specific lactate transporters. Intriguingly, in the absence of lactate permeases growth of *S. oneidensis* is allowed, albeit severally impaired. While these data indicate that lactate transporters are crucial for utilization of lactate as carbon and energy source, it manifests the presence of an auxiliary route (Fig. 7). This route may also works for lactate-based iron transport. Efforts to identify the route and to test this notion are underway.

*S. oneidensis* employs a large number of cytochromes *c* to sustain its diverse respiratory pathways, including those for efficient respiration of oxygen such as cytochromes *cbb*_3_ oxidase and *bc*_1_ complexes^29, 35^. Depletion of PutA hampers *S. oneidensis* to obtain iron from low-iron conditions, greatly impairing, if not abolishing, biosynthesis of cytochromes *c*. Despite this, aerobic growth is allowed in their absence, owing to the presence of cytochrome *bd*, an oxidase that is inferior to *cbb*_3_ in efficiency but plays an important role in combating various stresses^30, 42^. By analyzing nitrite susceptibility of the *putA* mutant, we found that cytochrome *bd* oxidase is likely produced at comparable levels, relative to that of the wild-type. Given substantial repression of the heme biosynthesis by iron shortage, it is conceivable that overall heme production is very low. When this is the case, cells apparently ensure production of cytochrome *bd* by blocking biosynthesis of cytochromes *c*, which confer respiratory diversity but not essentiality. We speculate that a concentration threshold for heme *b* must be met in order for biosynthesis of cytochromes *c* to commence.

## Electronic supplementary material

Supplemental Materials
