# Supplementary material for: Investigation of a spontaneous mutant reveals novel features of iron uptake in Shewanella oneidensis
Source: Sci Rep. 2017 Sep 18;7:11788. doi: 10.1038/s41598-017-11987-3 (PMC5603553; doi:10.1038/s41598-017-11987-3)
Supplement: Supplementary file 1 — Supplemental Materials [file 41598_2017_11987_MOESM1_ESM.pdf]

Supplemental materials of

Investigation of a spontaneous mutant reveals novel features of iron uptake in *Shewanella oneidensis*

Ziyang Dong,[†] Shupan Guo,[†] Huihui Fu, and Haichun Gao*

Institute of Microbiology and College of Life Sciences, Zhejiang University, Hangzhou, Zhejiang, 310058, China

Table S1 TonB-dependent receptors in *S. oneidensis*

Locus (Gene)	Length (a.a.)	Annotation
SO_0564	393	zinc-regulated TonB-dependent zinc receptor
SO_0630 (NosA)	668	TonB-dependent copper receptor
SO_0719	747	TonB-dependent hemoprotein receptor
SO_0737 (NicT)	692	TonB-dependent nickel receptor
SO_0798	720	TonB-dependent receptor
SO_0815 (BtuB)	653	TonB-dependent vitamin B12 receptor
SO_1102	683	TonB-dependent receptor
**SO_1156**	715	**TonB-dependent siderophore receptor**
SO_1309	1007	TonB-dependent receptor
SO_1482	815	TonB-dependent receptor
SO_1580	737	TonB-dependent haem/haemoglobin receptor
SO_1822	936	TonB-dependent receptor
SO_2427	853	ArgR-regulated TonB-dependent receptor
SO_2469	891	TonB-dependent receptor
SO_2523	943	TonB-dependent phytase receptor
SO_2715	733	thiamine-regulated TonB-dependent receptor
SO_2907	884	ArgR-regulated TonB-dependent receptor
**SO_3033 (PutA)**	730	**TonB-dependent ferric putrebactin siderophore receptor**
SO_3514	874	TonB-dependent chitooligosaccharide receptor
SO_3669 (HmuA)	697	TonB-dependent heme/hemoglobin receptor
**SO_3914**	730	**TonB-dependent siderophore receptor**
SO_4077	708	TonB-dependent receptor
**SO_4422**	724	**TonB-dependent ferric achromobactin receptor**
**SO_4516**	685	**TonB-dependent siderophore receptor**
**SO_4523 (IrgA)**	663	**iron-responsive TonB-dependent enterobactin receptor**
**SO_4743**	706	**TonB-dependent siderophore receptor**

Table S2. Sequence similarities between *E. coli* and *S. oneidensis* lactate permeases[a]

	LldP (551 a.a.)		GlcA (560 a.a.)	
	E-value	Identities (%)	E-value	Identities (%)
SO0827 (545 a.a.)	0	67	0	66
SO1522 (547 a.a.)	5e-21	27	1e-26	29

[a] *E. coli* LldP and GlcA are lactate/glycolate:H^+ symporters. Values are from BLASTp analysis.

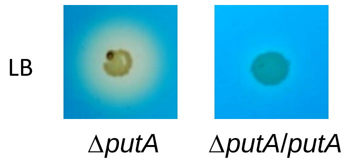

Figure S1. Siderophore production. The *putA* mutant carrying empty vector (Δ*putA*) or vector with *putA* (Δ*putA*/*putA*) were grown on LB agar plates for 24 hours. Siderophore was examined by the CAS assay.

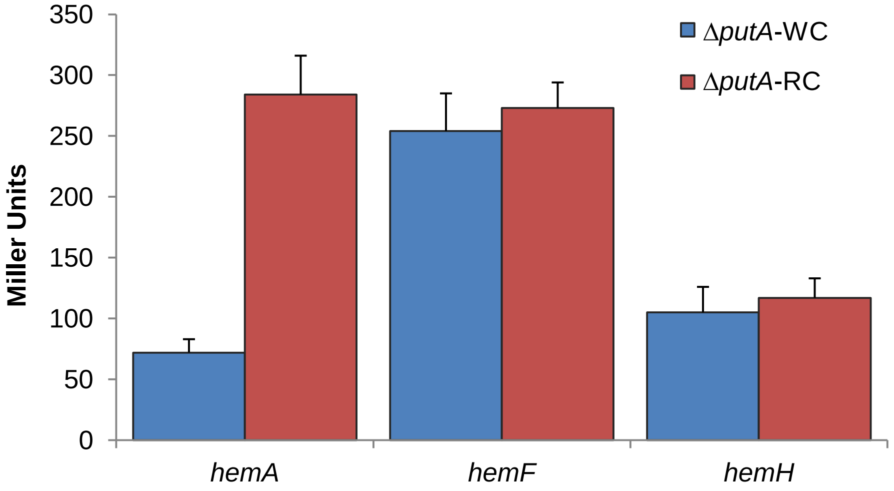

Figure S2. Expression of the representative *hem* genes in Δ*putA* WC and RC cells by *lacZ*-reporter. Cells of mid-log phase were prepared the same as in **Fig. 4B**. All experiments were performed in triplicate and error bars indicate the standard error of the mean.

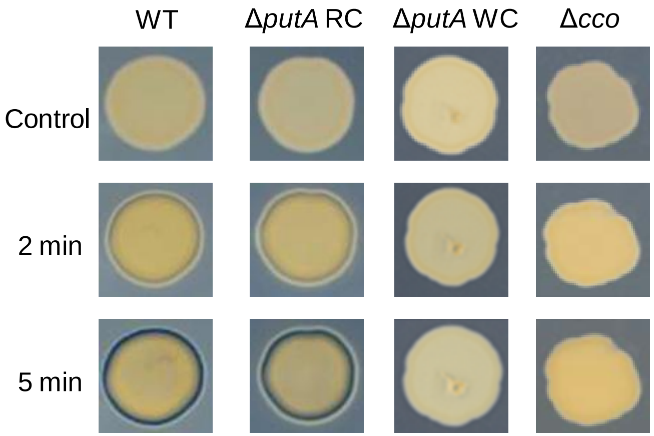

Figure S3. Cytochrome *cbb*$_3$ activity in indicated strains on LB plates by the Nadi assay. The method is based on the rapid formation of indophenol blue from colorless a-naphthol catalyzed by cytochrome *c* oxidase, using N',N'-dimethyl-p-phenylenediamine monohydrochloride as an exogenous electron donor. Photos were taken at indicated times after the reaction started. The wild-type and Δ*ccoN* strains serve as positive and negative controls.

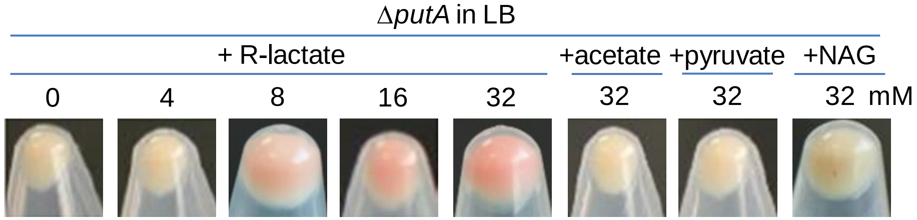

Figure S4. Effect of various carbon sources on the culture color of Δ*putA*. Cells were grown in LB with addition of one of indicated carbon sources at indicated concentrations. All experiments were performed at least three times and similar results were obtained.

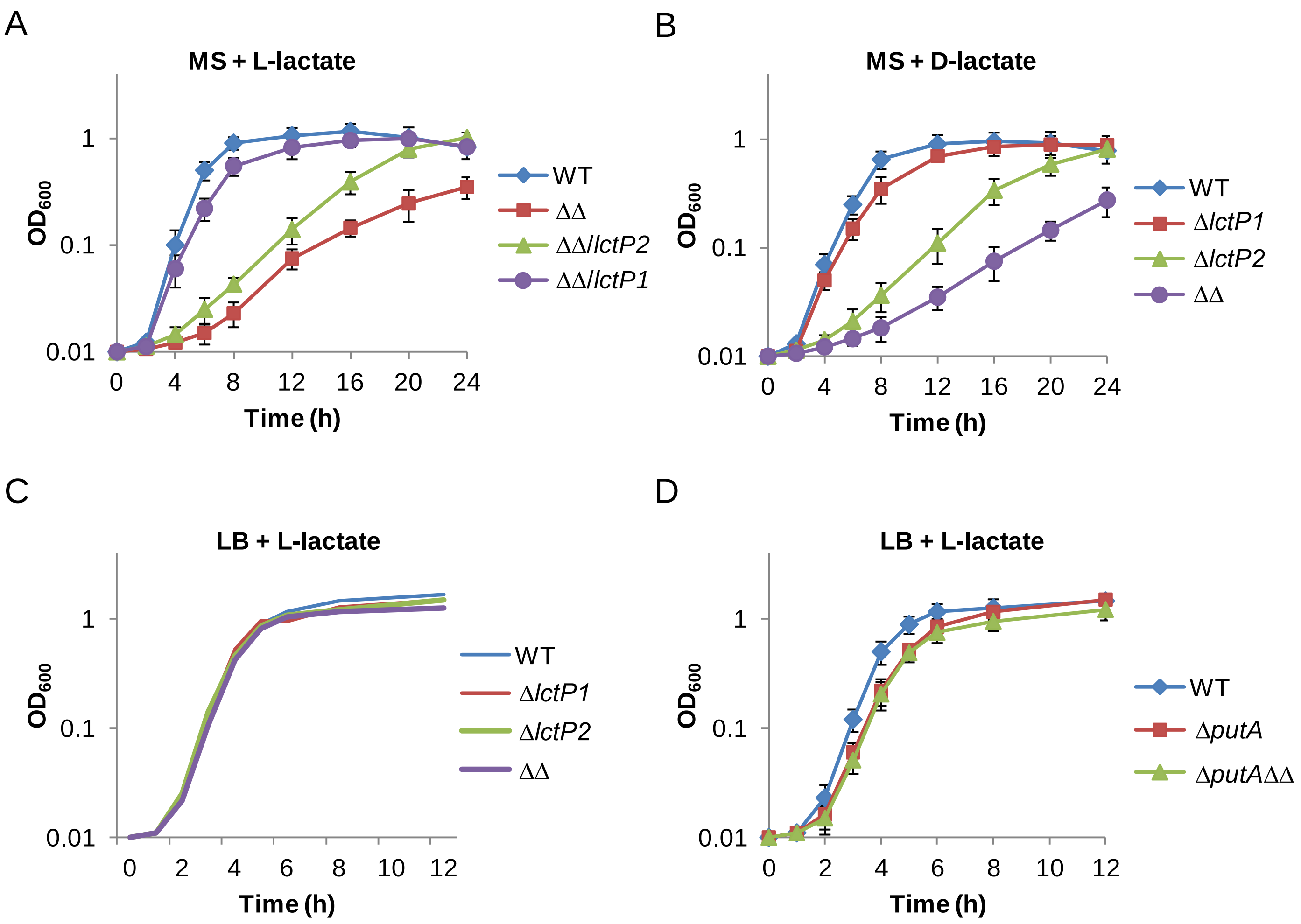

Figure S5. Growth of various *S. oneidensis* strains. A. Complementation of Δ*lctP1*Δ*lctP2* (ΔΔ, same in all panels) with either missing gene. Growth of indicated strains was recorded from MS containing 30 mM L-lactate. Expression of *lctP1* and *lctP2* was driven by Ptac with 0.1 mM IPTG. **B**. Growth of indicated strains in MS containing 30 mM D-lactate. **C.** Growth of indicated strains in LB containing 30 mM L-lactate. **D.** Growth of indicated strains in LB containing 30 mM L-lactate. Data are shown as mean ± SEM (standard error of the mean) from at least three experiments. In **C**, error bars were omitted for clarity.
